# Exploring the Anti-Chagas Activity of *Zanthoxylum chiloperone*’s Seedlings Through Metabolomics and Protein–Ligand Docking

**DOI:** 10.3390/plants14060954

**Published:** 2025-03-18

**Authors:** Ninfa Vera de Bilbao, Ryland T. Giebelhaus, Ryan P. Dias, Maria Elena Ferreira, Miguel Martínez, Lorea Velasco-Carneros, Seo Lin Nam, A. Paulina de la Mata, Jean-Didier Maréchal, Ahissan Innocent Adou, Gloria Yaluff, Elva Serna, Muriel Sylvestre, Susana Torres, Alicia Schinini, Ricardo Galeano, Alain Fournet, James J. Harynuk, Gerardo Cebrián-Torrejón

**Affiliations:** 1 Instituto de Investigaciones en Ciencias de la Salud (IICS), Universidad Nacional de Asunción, Asuncion 2169, Paraguay; meferpar@hotmail.com (M.E.F.); gloriayaluff@yahoo.com (G.Y.); elvsern@hotmail.com (E.S.); susitorres1@hotmail.com (S.T.); aschinini@gmail.com (A.S.); ricar.gal.08@gmail.com (R.G.); 2 The Metabolomics Innovation Centre (TMIC), Edmonton, AB T6G 2N4, Canada; rgiebelh@ualberta.ca (R.T.G.); dias1@ualberta.ca (R.P.D.); seolin@ualberta.ca (S.L.N.); delamata@ualberta.ca (A.P.d.l.M.); james.harynuk@ualberta.ca (J.J.H.); 3 Department of Chemistry, University of Alberta, Edmonton, AB T6G 2N4, Canada; 4 Laboratorio de Química, Laboratorio de Investigaciones Quimicas, Facultad de Ingenieria, Filial Ayolas, Universidad Nacional de Asunción, Centro de Investigaciones de Productos Naturales y de Síntesis, CIPRONAS, Ciudad de Ayolas 080202, Paraguay; miguelangelquimi@hotmail.com; 5 Instituto Biofisika (CSIC-UPV/EHU), Departamento de Bioquímica y Biología Molecular, Facultad de Ciencia y Tecnología, Universidad del País Vasco (UPV/EHU), 48940 Bilbao, Spain; loreavelasco22@gmail.com; 6 Insilichem, Departament de Química, Universitat Autònoma de Barcelona (UAB), Cerdanyola del Vallès, 08193 Barcelona, Spain; jeandidier.marechal@uab.cat; 7 Laboratoire COVACHIM-M2E EA 3592, Université des Antilles, 97157 Pointe-à-Pitre, France; adouinnocent1@gmail.com (A.I.A.); muriel.sylvestre@univ-antilles.fr (M.S.); gerardo.cebrian-torrejon@univ-antilles.fr (G.C.-T.); 8 IRD, Laboratoire de Pharmacognosie, Faculté de Pharmacie, Université Paris-Saclay, 92290 Châtenay-Malabry, France

**Keywords:** canthin-6-ones, chagas disease, medicinal plants, natural products, plant metabolomics, Rutaceae

## Abstract

This publication reports the controlled cultivation of *Zanthoxylum chiloperone* var. *angustifolium* Engl. (Rutaceae) in several growth substrates under controlled greenhouse conditions. This plant is well-known for its anti-Chagas (trypanocidal) activity, related to the presence of several β-carboline alkaloids. The metabolomic study of *Z. chiloperone* seedlings over two years of growth (2018–2020) was performed using comprehensive two-dimensional gas chromatography time-of-flight mass spectrometry (GC × GC-TOFMS). The canthin-6-one alkaloids, canthin-6-one and 5-methoxy-canthin-6-one, were putatively identified in *Z. chiloperone* extracts. Finally, in vitro and in silico studies of trypanocidal activity were performed, suggesting that canthin-6-one alkaloids could interact with the main pharmacological targets against *Trypanosoma cruzi*, cruzain protease, dihydroorotate dehydrogenase, lanosterol 14-alpha-demethylase, farnesyl diphosphate, and squalene synthases.

## 1. Introduction

Chagas disease (American trypanosomiasis) is an infectious disease caused by the parasite *Trypanosoma cruzi*, commonly found in the rural areas of Latin America. *Trypanosoma cruzi* thrives in the feces of members of the insect subfamily Triatominae, colloquially known as “kissing bugs” or “assassin bugs”. The parasite is transmitted to animals and humans when the insects feed on their blood and defecate infectious *T. cruzi* protozoa. The limitation of therapeutic strategies has been one of the biggest challenges in the fight against Chagas disease. Currently, no satisfactory treatment exists for Chagas disease, especially for the chronic stage [1]. Nifurtimox and benznidazole, developed in the 1970s, are still the only commercially available treatments with demonstrable efficacy for Chagas disease [2,3]. In fact, the only drug available in most Latin American countries is benznidazole. This drug produces significant side effects and has low efficacy for the chronic phase of the disease [4]. For this reason, it is urgent to find new solutions that are more effective and safer for the treatment of Chagas disease [5,6,7,8,9,10].

**Figure 1:**
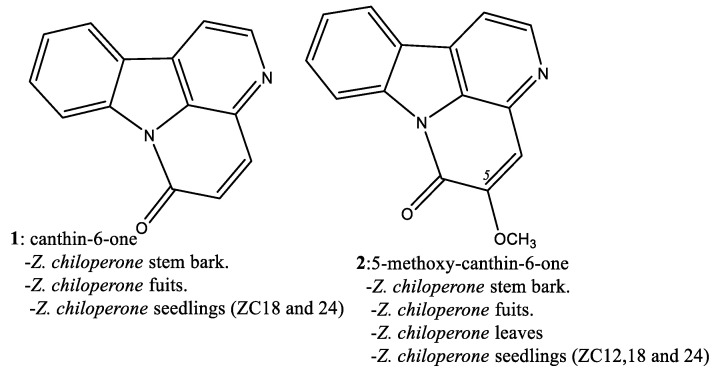
Structure of canthin-6-one alkaloids identified in *Zanthoxylum chiloperone*.

Interest in natural products chemistry, medicinal plants, and useful plant extracts has been growing. In the case of Chagas’ alternative therapies, several publications study the genus *Zanthoxylum* [11,12,13,14]. This genus comprises approximately 250 species worldwide, distributed mainly in subtropical and temperate regions. Many *Zanthoxylum* species are studied for their ethnopharmacological applications [15,16]. Recently, the anti-Chagas activity of *Zanthoxylum chiloperone* var. *angustifolium* Engl. (syn. *Fagara chiloperone* Engl. Ex Chod. & Hassl., syn. *Zanthoxylum caribaeum* Lam.) has been reported. *Z. chiloperone* is a Rutaceous plant growing in the Caribbean basin, the Eastern part of Paraguay and in the state of Parana, Brazil [17,18]. Currently, the natural abundance of *Z. chiloperone* has been drastically diminished by excessive harvest, the utilization of leaves in traditional remedies, and the destruction of the plants by the general population. The plants are generally considered impractical for human purposes, due to their many thorns and intense odor when burned as firewood (observed by authors). Furthermore, *Z. chiloperone* is difficult to cultivate, with a very low germination rate under natural growth conditions. The stems, bark, leaves, and various tissues of this Rutaceous plant contain several alkaloids of the β-carboline type, i.e., canthinone alkaloids (canthin-6-one (1) and 5-methoxycanthin-6-one (2); Figure 1) [11,12,13,14,19].

The presence of canthinones in several tissues of the adult tree of *Z. chiloperone* (bark, leaves, fruits, and roots) has been characterized previously (HPLC-UV-MS, cyclic voltammetry (CV)) [19,20]. These alkaloids, along with other secondary metabolites, may contribute to the observed beneficial properties of *Z. chiloperone* tissues. Canthinones are detectable in mature *Z. chiloperone*; at the time of this publication, there is no report describing seedling chemistry. A study of the seedlings using the metabolomics approach may yield a metabolite profile that corresponds to interesting pharmacological properties, as demonstrated in another *Zanthoxylum* species [21]. Comprehensive two-dimensional gas chromatography time-of-flight mass spectrometry (GC × GC-TOFMS) has been increasingly applied to untargeted plant metabolomics studies [22,23,24,25]. This methodology produces rich metabolite profiles which may further inform on the utilities of medicinal plants like *Z. chiloperone*. This work represents the first report of the controlled cultivation of *Z. chiloperone* and the metabolomic study of the seedlings over two years (2018–2020) using GC × GC-TOFMS. Also, in vitro and in silico studies of *Z. chiloperone*’s individual components were performed to assess their trypanocidal activity.

## 2. Materials and Methods

### 2.1. Seedling Production

The vegetative and germination multiplication of *Z. chiloperone* was carried out using several substrates (see Table S1). Seeds and cuttings were obtained from a wild plant of *Z. chiloperone* collected from the Piribebuy district of Paraguay. The cultivation technique used was described by Cordero and Boshier [26]. All agronomic activities of this project were carried out in the experimental field of the “Quiloperone Biological Experimental Center (QBEC)”, located in the “Compañía Cañada”. The field consists of four hectares of land (5 km from the city center of Piribebuy, Paraguay). The QBEC site includes a plant nursery and a research laboratory. One-half of the collected seeds were stored in a refrigerator at 4 °C and the other half at room temperature (approximately 25–35 °C) until use.

For the germination and vegetative propagation of the seedlings, six different growth substrates were used under controlled greenhouse conditions. Substrates included bovine manure, mulch, commercial fertilizer, washed sand, sawdust, and rice husk (Table S1). Seeds from both groups (stored refrigerated or at room temperature) were planted in each substrate. The seedlings required frequent watering during the first two years of growth. After two years, irrigation was applied as needed. Once germinated, the seeds were transferred to assay tubes and then to plastic pots; when they reached a height of approximately 25 to 50 cm, seedlings were transplanted to the experimental field.

### 2.2. Plant Material

The harvest of *Z. chiloperone* seedlings (ZC) was performed by manual pruning, the best period being the beginning of autumn (May) [25]. The seedlings were collected at three different growth stages: 12 months (ZC12), 18 months (ZC18), and 24 months (ZC24). A voucher specimen (MEF55) has been deposited at the “Herbarium of Chemical Sciences” Faculty, San Lorenzo, Paraguay. The identification was confirmed by the botanist of the herbarium (and validated by http://www.plantsoftheworldonline.org/ and www.theplantlist.org). The material harvested was obtained by pruning leaves and branches. Open shade drying was carried out by a dryer with air conditioning regulated for a maximum temperature of 40 °C. After drying, the branches and leaves were separated manually.

### 2.3. Extract Preparation

Once seedlings were dried, they were crushed to a micro-powder texture with a conventional mechanical mill (unmarked grinder). Subsequently, 1.5 kg of powder (particles size 500–1000 µm) of ZC12, 1 kg of ZC18, and 1.2 kg of ZC24 were basified with concentrated NH_4_OH (Sigma Aldrich, Oakville, ON, Canada), soaking the samples, and homogenizing the material until completely moistened. The moistened homogenate was allowed to dry and was macerated with excess dichloromethane (CH_2_Cl_2_ (Sigma Aldrich)) for a period of 30 days with stirring at least once per day (temperature 25 °C). Extraction was monitored by thin-layer chromatography (TLC) carried out on Merck Kieselgel silica gel plates (60F-254; Merck Millipore, Burlington, MA, USA) using ultraviolet (UV) light as the visualizing agent. Macerated extracts were filtered through a filter funnel with a 0.45 µm glass membrane and evaporated by means of a rotary evaporator. The yields of crude extracts were 340 g of ZC12 (yield 20%), 250 g of ZC18 (25%), and 290 g of ZC24 (24%).

### 2.4. Metabolomic Study

#### 2.4.1. Derivatization Procedure

Using a Rainin XLS Digital pipette (Mettler Toledo Inc., Mississauga, ON, Canada) with 200 μL filter tips (Froggabio Inc., Concord, ON, Canada), 50 μL of extracts were transferred to clean 2 mL GC vials (Chromatographic Specialties Inc., Brockville, ON, Canada). The extracts were dried under nitrogen at 50 °C using a 099A EV2412S Glass-Col Heated Analytical Evaporator (Cole-Parmer, Quebec City, QC, Canada) until a residue formed on the walls and bottoms of vials. A 100 μL aliquot of anhydrous HPLC grade toluene, (Sigma Aldrich) (dried with anhydrous sodium sulfate (Millipore Sigma, Oakville, ON, Canada)) was added to each of the samples. Toluene aliquots were dried under pre-purified nitrogen (Praxair Canada Inc., Edmonton, AB, Canada) at 40 °C to remove trace amounts of water. Dried residues were removed from the heating block and allowed to cool for 5 min. An aliquot (50 μL) of 20 mg/mL of methoxyamine hydrochloride (Sigma Aldrich) was added to each vial. Samples were returned to the heating block for 30 min at 60 °C to promote oximation. After oximation was complete, an aliquot (100 μL) of MSTFA (2,2,2-trifluoro-N-methyl-N-(trimethylsilyl)-acetamide) + 1%TMCS (chlorotrimethylsilanes) (Fisher Scientific, Edmonton, AB, Canada) was added to each vial; samples were incubated in the heating block for 30 min at 60 °C to allow complete silylation. An aliquot (100 μL) of each sample was transferred to glass insert vials and capped with GC vial caps containing polytetrafluoroethylene (PFTE) septa vials (Chromatographic Specialties Inc., Brockville, ON, Canada) prior to GC × GC-TOFMS analysis.

#### 2.4.2. GC × GC-TOFMS Method

The analyses were performed using a Leco Pegasus 4D GC × GC-TOFMS (LECO Corporation, St. Joseph, MI, USA) with a quad-jet liquid nitrogen thermal modulator. Liquid injections were performed using an MPS rail system (Gerstel Inc., Linthicum, MD, USA), and the inlet was a programmed-temperature vaporization inlet (CIS4, Gerstel Inc.). The first-dimension column was a 60 m × 0.25 mm × 0.25 μm Rxi-5SilMS and the second-dimension column was a 1.6 m × 0.25 mm × 0.25 μm Rtx-200MS (Chromatographic Specialties). Ultra-pure helium (5.0 grade; Praxair Canada Inc.) was used as the carrier gas, with a constant flow rate of 2.0 mL/min. The inlet was operated in splitless injection mode, using a glass-wool-filled inlet liner (Gerstel Inc.) and an injection volume of 1 μL. The inlet temperature started at 80 °C and was ramped to 250 °C within 1 min for all runs. The temperature program of the primary oven began at 80 °C (hold 4 min), followed by a ramp of 3.5 °C/min to 315 °C (hold 10 min). The secondary oven and modulator temperature offset were kept constant at +5 °C and +15 °C, respectively. The modulation period was 2.5 s. Mass spectra were collected at an acquisition rate of 200 Hz over a mass range of 40 to 800 m/z. The optimized detector voltage offset was 200 V with an electron impact energy of −70 eV. The ion source temperature was 200 °C with a transfer line temperature of 250 °C

### 2.5. Data Processing and Analysis

GC × GC-TOFMS data were processed using ChromaTOF^®^ (v.4.72; LECO). The baseline offset was set to 0.9 above the middle of the noise. The minimum S/N ratio for base and sub-peaks was set at 100, and the mass spectral match required for sub-peaks to be included in the auto-smoothed peak was set at 650. Expected peak widths throughout the entire chromatographic run were assumed to be approximately 10 s in the first dimension and 0.15 s in the second dimension. One region of each chromatogram was excluded from data processing, from 0.805 s to 1.14 s in the second dimension along the entire length of the separation in order to exclude siloxane peaks. Compounds were tentatively identified based on mass spectral (>700) and linear temperature-programmed retention index (LTPRI) matching (±20). All putatively identified compounds were identified according to the Metabolomics Standards Initiative (MSI) level 2. Mass spectra were searched against the NIST 2017 MS library (NIST, Department of Commerce; Gaithersburg, MD, USA) and Wiley (John Wiley & Sons, Inc., Hoboken, NJ, USA) 8th edition (WN08) MS library. LTPRI values were compared to those reported in PubChem and the NIST Chemistry WebBook online.

### 2.6. Biological Assays

#### 2.6.1. Cytotoxic Activity

Balb/c mice infected with *T. cruzi* (strain CL) were used. Macrophages were harvested from the abdominal cavity of Balb/c mice and seeded (1 × 10^5^ cells/well) in 24-well microplates with 100 μL of RPMI 1640 medium. The cells were allowed to attach for 24 h at 37 °C in 5% CO_2_. Thereafter, the medium was removed, and replaced with 200 μL of medium containing different concentrations of the plant extracts and incubated for an additional 24 h. Trypomastigotes were suspended in trypan blue solution at 0.4% and counted in a Neubauer chamber [27]. The results of the viability test were expressed as the number of a total of 10^5^ macrophages (Table S2). Each concentration was assayed three times. Culture medium (RPMI) and dimethylsulfoxide (DMSO; Sigma Aldrich) were used in each test as blanks.

#### 2.6.2. Trypanocidal Activity

Balb/c mice infected with *T. cruzi* (strain Epsilon) one week after infection were used. Blood was obtained by cardiac puncture using 3.8% sodium citrate as an anticoagulant in a 7:3 blood/anticoagulant ratio. The parasitaemia in infected mice ranged between 1 × 10^5^ and 5 × 10^5^ parasites per milliliter. Plant extracts were dissolved in cold dimethyl sulfoxide (DMSO (Sigma Aldrich^®^)) to a final concentration of 250 μg/mL. Aliquots of 10 μL of each extract of different concentrations were mixed in 96-well microtiter plates with 100 μL of infected blood containing 10^6^ parasites per mL. Infected blood and infected blood containing gentian violet at 250 μg/mL were used as controls. The plates were shaken for 10 min at room temperature and kept at 4 °C. Then, a 5 µL sample was placed on a slide and covered with a 22 × 22 mm coverglass for parasite counting. The lysis effect on mouse blood trypomastigotes was determined (Table 1) for extracts of *Z. chiloperone* at multiple µg/mL concentrations for triplicates [28,29]. The IC_50_ values were determined with Origin software 8 data Analysis Graphic Sofware (OriginLab Corporation)—using dose–response curves. IC_50_ was calculated by the Finney D J. Probit analysis.

The following formula (Equation (1)) was used to determine the percentage of lysis (death) [30]:

(1) $$\%\;lysis=\frac{N_{pps}-N_{pce}}{N_{pps}}*\;100$$

where

*N_pps_* indicates the number of parasites per mL of physiological solution.

*N_pce_* indicates the number of parasites per mL of culture with the extract.

0% indicates null sensitivity of the parasite to the drug or extract.

1–25% indicates low sensitivity of the parasite to the drug or extract.

26–50% indicates the average sensitivity of the parasite to the drug or extract.

51–75% indicates acceptable sensitivity of the parasite to the drug or extract.

76–100% indicates high sensitivity of the parasite to the drug or extract.

### 2.7. Computational Methods

To evaluate the most probable molecular targets of canthinones (canthin-6-one and 5-methoxy-canthin-6-one), a molecular modeling study was performed. It consisted of a two-step protein–ligand docking for the two compounds to six known important targets of *T. cruzi*: cruzain protease (PDB ID: 3KKU), dihydroorotate dehydrogenase (PDB ID: 2DJX), Lanosterol 14-alpha-demethylase (PDB ID: 2WX2), dihydrofolate reductase-thymidylate (PDB ID: 3IRM), farnesyl diphosphate synthase (PDB ID: 4E1E), and squalene synthase (PDB ID: 3WCC) [31]. For some of these proteins, several crystal structures are available in the Protein Data Bank, each with different ligands bound. For these systems, the structures that are bound to ligands with the highest structural similarity to canthinones were selected.

To ascertain the main site of canthinone interaction and to find possible differences with the crystallographic ligands, a blind docking was carried out using GaudiMM [32] with 3 objectives: LigScore, clashes and hydrogen bonds. Once the most probable binding pocket for each protein was found, a second round of protein–ligand dockings was performed on those regions using the program Gold 5.8 and ChemScore as a scoring function [33,34].

## 3. Results

### 3.1. Seedling Production and Extraction Work

The substrates with the best yields were the washed sand and the commercial fertilizer with germination rates of 6.5% and 5%, respectively. The other substrates yielded the following germination rates: bovine manure: 4.5%; mulch: 2%; sawdust: 1.5%; and rice husk: 0%. The total percentage of germinated seeds compared to sow seeds is 2.5%. These results confirm that this plant species has a very low germination rate (Table S1). Sapling formation was slow (4–5 months), and the proper season for transplanting to the cultivation field was during the spring and summer months. Comparing the groups of seeds stored at room temperature with those stored in the refrigerator at 4 °C, the seeds stored in the cold presented higher germination rates.

### 3.2. Metabolomics Analysis

The metabolite analysis was performed on *Z. chiloperone* seedlings at different developmental stages: 12 (ZC12), 18 (ZC18), and 24 (ZC24) months. Two-dimensional chromatograms of the three samples are shown in Figure S3 presenting very similar profiles. Each contour plot is represented with the first-dimension retention time in seconds on the x-axis and the second-dimension retention time in seconds on the y-axis, and peak intensities are depicted using a color scale, on the z-axis. The metabolite profiles for each seedling age contained approximately 6000 detected peaks, each represented as a blob in the 2D chromatogram (Figure S2) per sample, using an S/N of 100. Among the approximately 6000 peaks, various families of compounds were found across all samples using the ChromaTOF^®^ (Leco Corporation, St. Joseph, MI, USA) scripting tool [35], including carbohydrates, alkanes, aldehydes, alcohols, ketones, amino acids, terpenoids (mono-, sesqui-, di-, tri-), free fatty acids, esters, sterols, and tocopherols (Table S3). Two canthinone alkaloids were putatively identified, as shown in Figure S2B. Zoomed-in extracted ion chromatograms, using mass channels of m/z 220 and 250 for canthin-6-one and 5-methoxy-canthin-6-one, respectively, are shown in Figures S1 and S2. The acquired mass spectra along with the matched library spectra for the two canthinone alkaloids are also shown in Figures S4 and S5.

### 3.3. Results of Cytotoxicity and Anti-Trypanosoma cruzi Activity In Vitro

Canthine-6-one (Figure S1) and 5-methoxy-canthin-6-one (Figure S2) were putatively identified by mass spectral and retention index matching.

Each extract was evaluated for cytotoxicity (Table S2) and anti-*T. cruzi* activity (Table 1). No extracts at a concentration of 50 or 100 µg/mL were determined to be cytotoxic.

### 3.4. Computational Analysis

First, a blind docking of the two canthinone derivatives, canthin-6-one and 5-methoxy-canthin-6-one, was performed on six proteins of *Trypanosoma cruzi*: cruzain protease (PDB ID: 3KKU), dihydroorotate dehydrogenase (PDB ID: 2DJX), Lanosterol 14-alpha-demethylase (PDB ID: 2WX2), dihydrofolate reductase-thymidylate (PDB ID: 3IRM), farnesyl diphosphate synthase (PDB ID: 4E1E), and squalene synthase (PDB ID: 3WCC) [36]. The calculations showed that the best binding orientations are found in the same pockets that have been characterized to bind the experimental ligands, thus suggesting the possibility of similar effects [37]. To better understand the interactions of the canthinones with the target proteins, a second set of calculations was performed. This iteration focused on accurately exploring the binding site identified in the blind docking. The docking results (i.e., Chemscore) are close to 30 units, a magnitude generally associated with good predicted binding affinity for protein–ligand complexes (Table 2).

**Figure 2:**
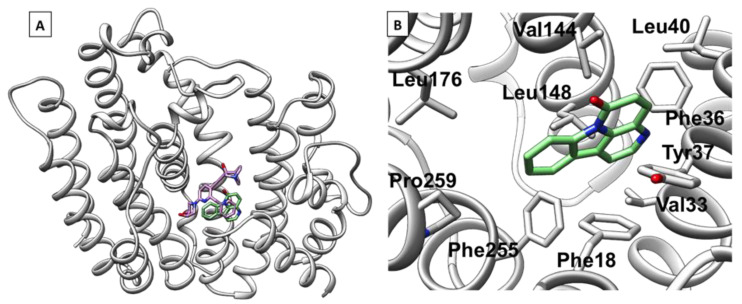
The potential binding pocket of squalene synthase (PDB ID: 3WCC) after the docking analyses were performed using Gold. (**A**) Best docking result of canthin-6-one (green) superpositioned with the crystal structure of squalene synthase (light gray) in complex with (3R)-3-({2-benzyl-6-[(3R,4S)-3-hydroxy-4-methoxypyrrolidin-1-yl]pyridin-3-yl}ethynyl)-1-azabicyclo [2.2.2]octan-3-ol (pink), a non-covalent inhibitor of this protein [37]. (**B**) Best docking of canthin-6-one with the crystal structure of squalene synthase.

Two proteins that are the most promising *T. cruzi* targets of canthinones were identified, and their docking results are given in Figure 2A,B.

## 4. Discussion

The current study aimed to extract useful secondary metabolites of *Z. chiloperone*, profile the extracts’ various chemical classes and assess cytotoxicity and anti-*T. cruzi* activity in vitro and in silico.

After the extraction process, the crude yields of extracts were 20% of ZC12, 25% of ZC18, and 24% of ZC24. These yields are comparable with the ones described in the literature for this species [6,7,8,9].

GC × GC is a powerful analytical technique which facilitates the separation of thousands of compounds using two different columns, allowing for the detection and identification of peaks which cannot be resolved by traditional one-dimensional GC (1D-GC). Additionally, GC × GC provides superior sensitivity, resolution, and selectivity over 1D-GC, providing purer analyte to the mass spectrometer, yielding more pure mass spectra to assist in putative identification at lower concentrations. When dealing with highly complex chromatograms containing thousands of metabolites, with abundances often spanning many orders of magnitude, GC × GC proved especially valuable, as demonstrated by the metabolite profiles of *Z. chiloperone* at various developmental stages. The technique’s ability to detect and identify trace amounts of canthinone alkaloids (Figure S2), despite the challenges posed by the complexity of the sample matrix, demonstrates its effectiveness in the metabolomic analysis of natural products.

Interestingly, only 5-methoxy-canthin-6-one was detected in the youngest seedling. As time went on, both canthinones were detected at 18 months. The three extracts presented approximately 6000 detected peaks per chromatogram. Tentatively identified compounds were selected from each chemical family and are provided in Table S3.

The activity of the extracts (Table 1) was evaluated with the trypomastigote form of the parasites at concentrations of 250, 100, 50, and 25 µg/mL; in these conditions, the extracts of 12 months produced 63% of lysis of the parasites corresponding an IC_50_ of 119 µg/mL, with the extract of 18 months, the reduction in the number of parasites was of 59% with an IC_50_ of 141 µg/mL. With the extract of 24 months, the reduction in the number of parasites was f 77%, with an IC_50_ of 71 µg/mL.

In the cytotoxicity tests, extracts of *Z. chiloperone* were evaluated at concentrations of 100, 50, and 25 µg/mL at 12, 18, and 24 months (Table S2). In the results, there is no evidence of cytotoxic action against macrophage cells, compared to the negative control, which has a viability of 100%.

Two proteins of all modeled proteins were predicted to have the highest affinity for the natural products; in particular, canthin-6-one is predicted to have the highest binding affinity for the squalene synthase and lanosterol 14-alpha-demethylase. A similar trend is observed for the interactions between the derivative compounds and the same two proteins. Both canthinone derivatives tend to be only slightly displaced from the original ligands present in the X-ray structure of squalene synthase (Figure 2A). The displacement was attributed to a higher planarity and aromaticity of canthinones with respect to the crystallographic structures. Focusing on these two systems, the docking pose analysis indicates that the interaction between squalene synthase and canthin-6-one is mostly stabilized by hydrophobic interactions between the aromatic rings of the compound and the hydrocarbon chains of non-polar residues in the binding pocket (Figure 2B). These interactions are largely maintained with 5-methoxy-canthin-6-one. For squalene synthase, the location of the canthinone derivatives is predicted to be the same binding site as those observed in X-ray structures. This shows that the natural compounds could have the convenient chemical properties to bind at the main binding site of the enzyme [37].

Current knowledge of CYP450 dynamics and substrate binding reveals that the possible rearrangement or binding of several molecules could occur to assist the inhibition process. Taken together, these docking analyses suggest a potential therapeutic route for canthin-6-one and 5-methoxy-canthin-6-one through the inhibition of six major target proteins of *T. cruzi*. Of the six targets, squalene synthase, followed by lanosterol 14-alpha-demethylase, seem to be the most promising targets for further exploration.

## 5. Conclusions

The present manuscript highlights the potential of *Z. chiloperone* seedlings as an alternative Chagas disease therapy. The germination, vegetative propagation, and harvest of *Z. chiloperone* seedlings was optimized; seedlings were obtained for extraction by manual pruning in the beginning of autumn in Paraguay. The vegetative and germination multiplication of *Z. chiloperone* was carried out under several controlled greenhouse conditions with six different substrates. Substrates included bovine manure, mulch, commercial fertilizer, washed sand, sawdust, and rice husk; washed sand and commercial fertilizers are the best options. Once collected, the seedlings were extracted with dichloromethane and their anti-*T. cruzi* activity was studied in vitro. The extracts demonstrated interesting activity against *T. cruzi* coupled with low toxicity to host cells. A concentration of 250 µg/mL of *Z. chiloperone* seedling extract at 24 months yielded higher sensitivity, in agreement with previous results from the literature with wild plants (78% sensitivity) [6]. These results may suggest that a 24-month-old seedling could be cultivated and used as a raw material in the production of phytomedicines. A metabolomic study of seedling extracts demonstrated the presence of known anti-*T. cruzi* active compounds, specifically canthin-6-one alkaloids: canthin-6-one and 5-methoxy-cantin-6-one. Finally, the potential of these alkaloids was explored in silico by molecular modeling, predicting that the studied canthinone alkaloids could possibly interact with the main targets against *T. cruzi* (cruzain protease, dihydroorotate dehydrogenase, lanosterol 14-alpha-demethylase, farnesyl diphosphate, and squalene synthases). Protein docking analysis implies that these compounds are potential modulators of squalene synthase and, to some extent, lanosterol 14-alpha-demethylase. These protein targets are predicted to have the highest affinity with canthin-6-one, suggesting that the seedlings of *Z. chiloperone* could serve as a sustainable and durable route for canthinone production. Future work should focus on *Z. chiloperone* cultivation, and further studies are now underway to evaluate their in vivo study to confirm it as a potential alternative Chagas disease treatment of the compounds produced by this method.

## Figures and Tables

**Table 1:** Comparison of the (A) activity of the extracts against the parasite and (B) IC_50_ values for extracts of 12, 18 and 24-month-old *Z. chiloperone* seedlings.

In Vitro Assays at Different Concentrations of *Zanthoxylum chiloperone* Against Trypomastigotes of *Trypanosoma cruzi*.* and Cytotoxicity in Peritoneal Murine Macrophages.						
Concentrations of Extract ^#^ (µg/mL)	% of Lysis Trypomastigotes	% of Lysis Murine Macrophages	% of Lysis Trypomastigotes	% of Lysis Murine Macrophages	% of Lysis Trypomastigotes	% of Lysis Murine Macrophages
	12 Months Old IC_50_ ** = 119 ± 10 µg/mL		18 Months Old IC_50_ ** = 141 ± 14 µg/mL		24 Months Old IC_50_ ** = 71 ± 8 µg/mL	
250	63 ± 5	ND ***	59 ± 7	ND	77	ND
100	54 ± 5	3 ± 1	48 ± 5	4 ± 1	59 ± 8	3 ± 1
50	27 ± 3	0 ± 1	30 ± 3	1 ± 1	36 ± 6	1 ± 1
25	0 ± 2	ND	0 ± 1	ND	23 ± 5	ND

* Control positive gentian violet, 100% lysis. ** IC_50_, minimum dose to inhibit 50% of parasites. *** ND = not determined. ^#^ Each concentration was assessed in triplicate.

**Table 2:** ChemScores for docking results for each protein and compound performed using Gold.

Canthinone Derivative	Cruzain Protease (PDB ID: 3KKU)	Dihydroorotate Dehydrogenase (PDB ID: 2DJX)	Lanosterol 14-Alpha-Demethylase (PDB ID: 2WX2)	Dihydrofolate Reductase-Thymidylate (PDB ID: 3IRM)	Farnesyl Diphosphate Synthase (PDB ID: 4E1E)	Squalene Synthase (PDB ID: 3WCC)
1: canthin-6-one	25.75	27.2	30.37	28.11	23.6	33.65
2: 5-methoxy-canthin-6-one	23.82	25.38	29.1	28.77	24.27	32.56

## Data Availability

The data presented in this study are available in the article and the Supplementary Information.

## Supplementary Materials

The following supporting information can be downloaded at: https://www.mdpi.com/article/10.3390/plants14060954/s1, Table S1: Protocols for seedlings production; Table S2: Cytotoxicity evaluation of 12,18 and 24-month-old (ZC12, ZC18 and ZC24 respectively) *Z. chiloperone* seedling extracts; Table S3: Tentatively identified compounds derivation protocol scripting and their retention indices (RI) as examples of metabolites present in *Z. chiloperone* seedlings; Figure S1: Chromatograms of canthin-6-one (1) using mass channel 220. A. ZC12, B. ZC18, C. ZC24. Horizontal axes represent first dimension retention time (s) and vertical axes represent second dimension retention time (2); Figure S2: Chromatograms of 5-methoxy-canthin-6-one (2) using mass channel 250. A. ZC12, B. ZC18, C. ZC24. Horizontal axes represent first dimension retention time (s) and vertical axes represent second dimension retention time (2); Figure S3: GC×GC-TOFMS contour total ion chromatograms obtained from each sample using the same colour scale for z-axis. A. ZC12; B. ZC18; C. ZC24. The horizontal axes represent the first dimension retention times (s) and vertical axes represent the second dimension retention times (s). In B, alkaloids canthin-6-one (1) and 5-methoxy-canthin-6-one, 5-methoxy (2) have been labeled; Figure S4: Experimental and library mass spectra of canthin-6-one (1). Vertical axis is relative abundance and horizontal axis is acquired *m/z* range; Figure S5: Experimental and library mass spectra of 5-methoxy-canthin-6-one (2). Vertical axis is relative abundance and horizontal axis is acquired *m/z* range.

## Abbreviations

Balb/mice	albino laboratory-bred strain house mice
CYP450	cytochrome 450
DMSO	dimethyl sulfoxide
GC	tgas chromatography
HPLC	high performance liquid chromatography
MPS	multi purpose sampler
NIST	National Institute of Science and Technology, USA
PDB	protein data bank
RPMI	Roswell Park Memorial Institute medium
Strain CL	clone Brener
