# Supplementary material for: Exploring the Anti-Chagas Activity of Zanthoxylum chiloperone’s Seedlings Through Metabolomics and Protein–Ligand Docking
Source: Plants (Basel). 2025 Mar 18;14(6):954. doi: 10.3390/plants14060954 (PMC11946004; doi:10.3390/plants14060954)
Supplement: Supplementary file 1 [file plants-14-00954-s001.zip › plants-3487691-supplementary.pdf]

Electronic Supplementary Information for:

Exploring the Anti-Chagas Activity of *Zanthoxylum chiloperone*'s Seedlings Through Metabolomics and Protein–Ligand Docking

Table S1. Protocols for seedlings production.

**Protocol 1: *Z. chiloperone* seeds sown in the month of October 2016**			
**Substrate**	**Number of seeds sown**	**Number of germinated seeds**	**Germination (%)**
Bovine manure	200	9	4.5
Mulch	200	4	2%
Commercial fertilizer	200	10	5 %
Sawdust	200	3	1.5 %
Rice husk	200	0	0 %
Washed sand	200	13	6.5 %
**Total**	**1200**	**39**	**3.25 %**
**Protocol 2: *Z. chiloperone* seeds sown in the month of January 2017**			
**Substrate**	**Number of seeds sown**	**Number of germinated seeds**	**Germination (%)**
Washed sand	200	13	6.5 %
Washed sand	200	0	0 %
Manure	200	2	1 %
Manure	200	5	2.5 %
Commercial fertilizer	200	1	0.5 %
Commercial fertilizer and manure (50:50)	200	2	1 %

**Total**	**1200**	**23**	**1.9 %**
**Protocol 3: *Z. chiloperone* seeds sown in the month of January 2017**			
**Substrate**	**Number of seeds sown**	**Number of germinated seeds**	**Germination (%)**
Washed sand	300	2	0.6 %
Washed sand	300	6	2 %
**Total**	**600**	**8**	**1.3 %**

Note: The difference between the three protocols is the number of seeds planted and the planting season.

Table S2. Cytotoxicity evaluation of 12,18 and 24-month-old (ZC12, ZC18 and ZC24 respectively) *Z. chiloperone* seedling extracts.

**Percentage ± SD of mouse peritoneal macrophage lysis.**				
**Conc. Extract 100 µg/mL**	**Conc. Extract 50 µg/mL**	**Conc. Extract 25 µg/mL**	**Control Pos.***	**Control Neg.***
**Cytotoxicity evaluation of extracts of 12-month-old *Z .chiloperone* seedlings (ZC12)**				
Alive* : 97± 1	A*: 100	A*:100	A*: 0	A*: 100
Dead*3 ± 1	D*: 0	D*: 0	D*:100	D*: 0
**Cytotoxicity evaluation of extracts of 18-month-old *Z .chiloperone* seedlings (ZC18)**				
Alive* : 96± 1	A*: 99	A*:99	A*: 1	A*: 99
Dead*4 ± 1	D*:1	D*: 1	D*:99	D*: 1
**Cytotoxicity evaluation of extracts of 24-month-old *Z.chiloperone* seedlings (ZC24)**				
Alive* : 97± 1	A*: 99	A*:99	A*: 1	A*: 98
Dead*3 ± 1	D*:1	D*: 1	D*:99	D*: 2

Control Neg*:Macrophage+RPMI medium   Control Pos*:Macrophage+DMSO A: alive D:dead

Table S3. Tentatively identified compounds derivation protocol scripting and their retention indices (RI) as examples of metabolites present in *Z. chiloperone* seedlings**.**

Class	Compound	Experimental RI	Library RI
Alcohols	1-Dodecanol#	1567.3	1559.6
	1-Tridecanol#	1664.3	1661.4
	1-Tetradecanol#	1762.3	1760
	1-Pentadecanol#	1860.7	1856.4
	1-Hexadecanol#	1958.4	1955.1
	1-Heptadecanol#	2050.7	2054
	1-Pentacosanol#	2822.1	2826
	1-Triacontanol#	3330.4	3333.9
Aldehydes	Nonanal	1107.6	1106
	Benzaldehyde	984.42	980
	Decanal	1221.7	1227
	Syringaldehyde#	1759.7	N/A
n-Alkanes*	C10-30	N/A	N/A
Amides	Neoherculin	2277.0	N/A
Amino acids	Serine#	1355.7	1368
	Proline#	1292.9	1293.8
	Alanine#	1098.2	1095.3
	Tyrosine#	1934.3	1928.5
Aromatic	*o*-Cymene	1023.5	1023

Carbohydrates	Fructose[#]	1875.0	1875
	Galactose[#]	1889.3	1897.6
	Glucose[#]	1908.4	1926
	Turanose[#]	2788.4	N/A
	Melibiose[#]	3227.9	N/A
	Maltose[#]	3322.7	N/A
	Ribitol[#]	1714.4	1710
Free Fatty Acids[*,#]	C6-C24	N/A	N/A
Fatty Acid Methyl Esters[*]	C6-C24	N/A	N/A
Fatty Acid Ethyl Esters	Ethyl octadecanoate	2214.8	2207
Isopropyl Esters	Isopropyl tetradecanoate	1823.7	1823
Ketones	2-Heptadecanone	1902.5	1902
	Hexahydropseudoionone	1404.4	1404
	6-Methyl-2-heptanone	967.95	965
Sterols	Campesterol[#]	3686.2	N/A
	β-Sitosterol[#]	3775.7	N/A
	Lupeol[#]	3696.7	N/A
	Stigmasterol[#]	3709.8	N/A
Terpenoids	D-Limonene	1033.5	1035
	Nerolidol[#]	1677.3	1691
	Neral	1243.2	1244

	Linalool[#]	1230.6	1227.4
	trans-β-Ionone	1484.5	1486
	Phytol[#]	2147.9	2162.5
	β-Eudesmol[#]	1752.8	1760
	γ-Terpinene	1061.4	1061
	Farnesyl acetone	1913.1	1913
	trans-β-Ocimene	1047.5	1044
	β-Caryophyllene oxide	1593.6	1581
	α-Ylangene	1352.3	1352
	β-Myrcene	993.03	993
Tocopherols	α-Tocopherol[#]	3551.7	N/A
	γ-Tocopherol[#]	3391.1	N/A
	δ-Tocopherol[#]	3275.3	N/A

[*] Compounds presented as a range of incrementally increasing carbon number since the homologous series was detected.

[#] PubChem and the NIST Chemistry WebBook were consulted to obtain library retention indices. Retention indices of denoted compounds listed herein may refer to their O-methyloxime and/or TMS derivatives due to the sample derivatization protocol.

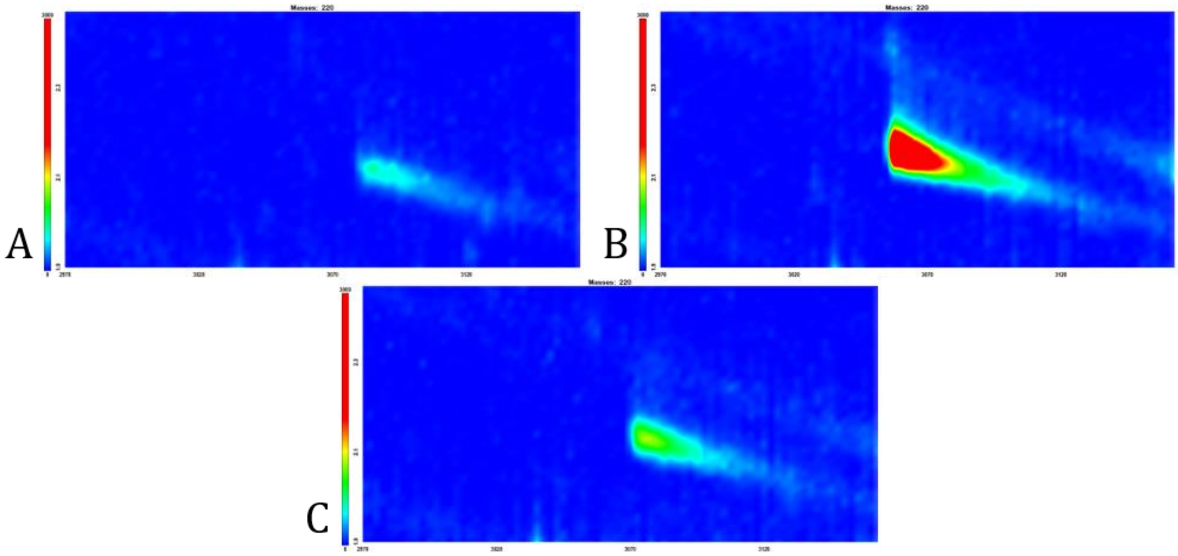

Figure S1. Chromatograms of canthin-6-one (**1**) using mass channel 220. A. ZC12, B. ZC18, C. ZC24. Horizontal axes represent first dimension retention time (s) and vertical axes represent second dimension retention time (2)**.**

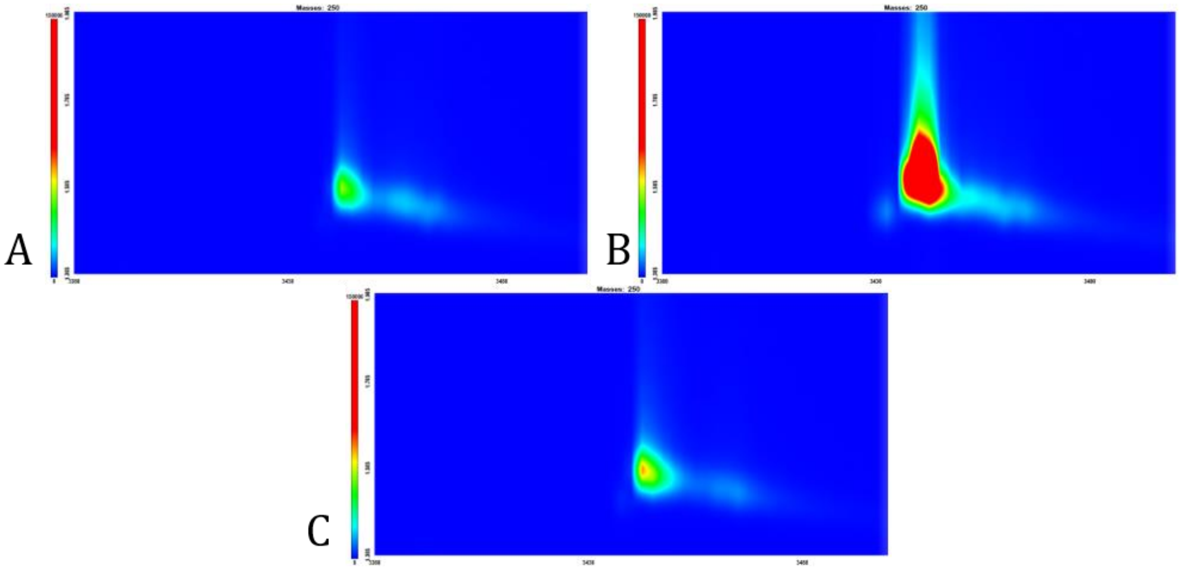

Figure S2 Chromatograms of 5-methoxy-canthin-6-one (**2**) using mass channel 250. A. ZC12, B. ZC18, C. ZC24. Horizontal axes represent first dimension retention time (s) and vertical axes represent second dimension retention time (2)**.**

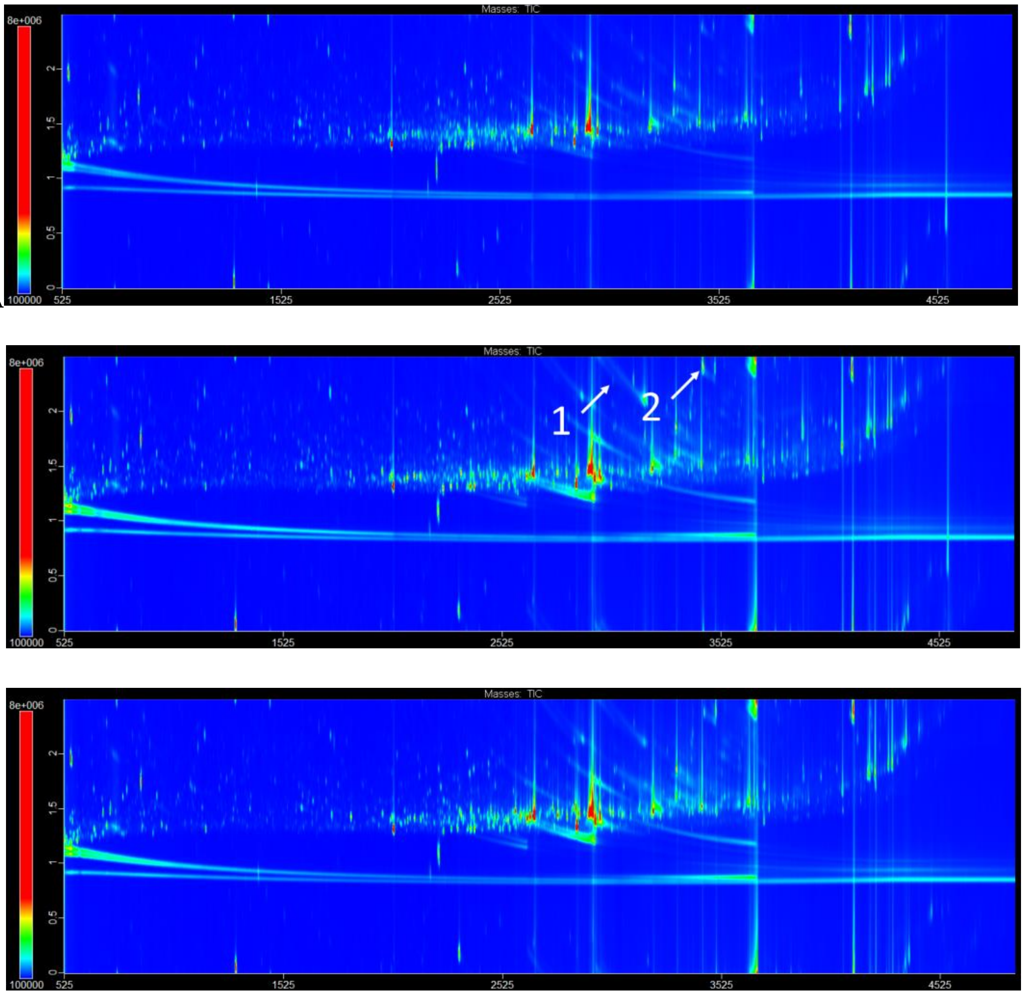

A B C

Figure 3S: GC×GC-TOFMS contour total ion chromatograms obtained from each sample using the same colour scale for z-axis. A. ZC12; B. ZC18; C. ZC24. The horizontal axes represent the first dimension retention times (s) and vertical axes represent the second dimension retention times (s). In B, alkaloids canthin-6-one (1) and 5-methoxy-canthin-6-one, 5-methoxy (2) have been labeled.

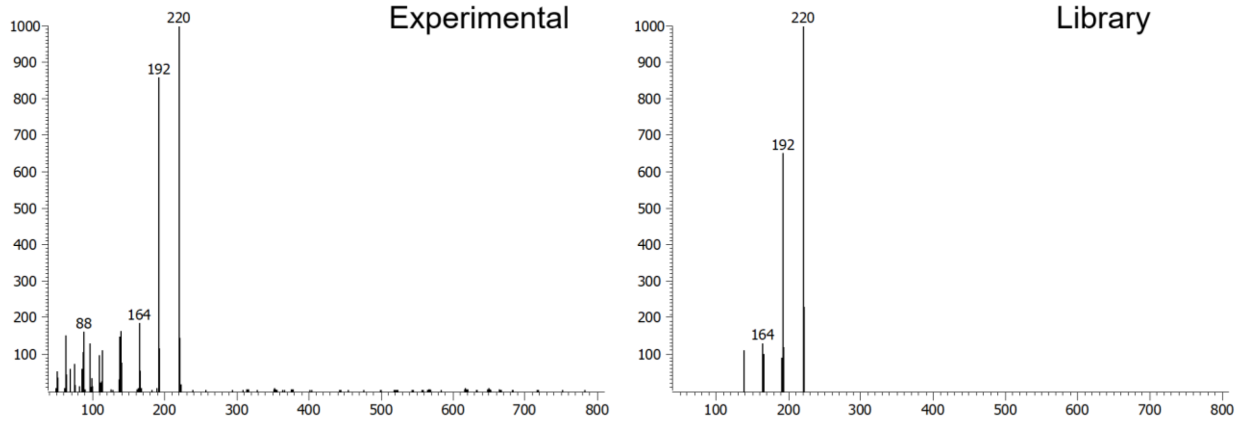

Figure S4. Experimental and library mass spectra of canthin-6-one (**1**). Vertical axis is relative abundance and horizontal axis is acquired *m/z* range.

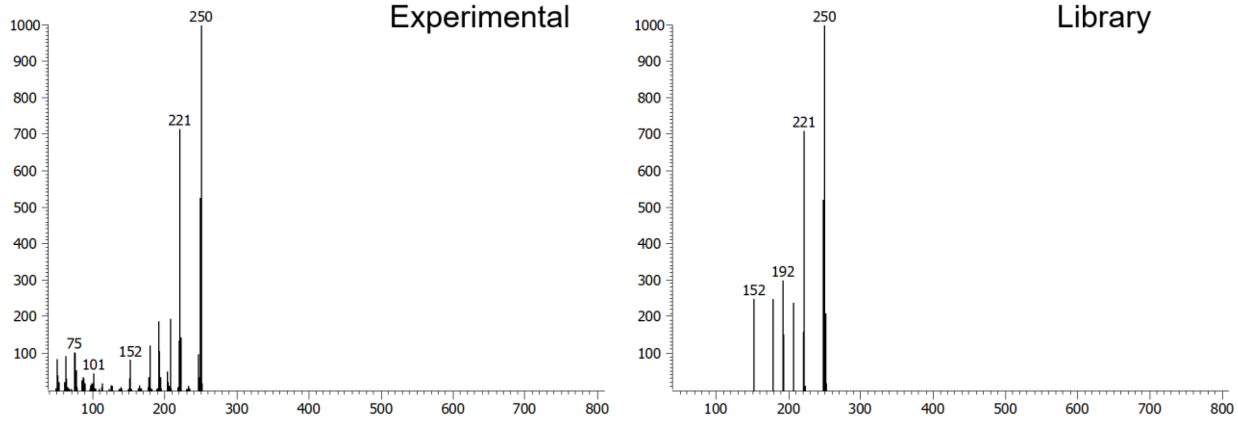

Figure S5. Experimental and library mass spectra of 5-methoxy-canthin-6-one (**2**). Vertical axis is relative abundance and horizontal axis is acquired *m/z* range.
